# Contractile and Elastic Ankle Joint Muscular Properties in Young and Older Adults

**DOI:** 10.1371/journal.pone.0015953

**Published:** 2011-01-11

**Authors:** Christopher J. Hasson, Ross H. Miller, Graham E. Caldwell

**Affiliations:** Biomechanics Laboratory, Department of Kinesiology, University of Massachusetts Amherst, Amherst, Massachusetts, United States of America; University Hospital Vall d'Hebron, Spain

## Abstract

The purpose of this study was to investigate age-related differences in contractile and elastic properties of both dorsi- (DF) and plantarflexor (PF) muscles controlling the ankle joint in young and older adults. Experimental data were collected while twelve young and twelve older male and female participants performed maximal effort isometric and isovelocity contractions on a dynamometer. Equations were fit to the data to give torque-angle (*Tθ*) and torque-angular velocity (*Tω*) relations. Muscle series-elasticity was measured during ramped dynamometer contractions using ultrasonography to measure aponeurosis extension as a function of torque; second order polynomials were used to characterize the torque-extension (*TΔL*) relation. The results showed no age differences in DF maximal torque and none for female PF; however, older males had smaller maximal PF torques compared to young males. In both muscle groups and genders, older adults had decreased concentric force capabilities. Both DF and PF *TΔL* relations were more nonlinear in the older adults. Older PF, but not DF muscles, were stiffer compared to young. A simple antagonism model suggested age-related differences in *Tθ* and *Tω* relations would be magnified if antagonistic torque contributions were included. This assessment of static, dynamic, and elastic joint properties affords a comprehensive view of age-related modifications in muscle function. Although many clinical studies use maximal isometric strength as a marker of functional ability, the results demonstrate that there are also significant age-related modifications in ankle muscle dynamic and elastic properties.

## Introduction

The functional capabilities of the dorsi- (DF) and plantarflexor (PF) muscles controlling the ankle joint are important in many activities of daily living. Age-related degradations of muscular properties such as a decline in maximal isometric ankle torque can impact these activities [1]. However, maximal isometric torque capability is only one of several joint properties that may change with advancing age. Ankle torque production also depends on the joint position or angle (the torque-angle relation; *Tθ*), the joint angular velocity (the torque-angular velocity relation; *Tω*), and the series elasticity (the torque-extension relation; *TΔL*) of the muscles crossing the joint. Together, these three relations reflect the active contractile and elastic properties of the muscles controlling the ankle joint.

To date, studies investigating age-related changes in DF and PF joint *Tθ* relations have produced equivocal results. Lanza et al. [2] have reported an angle-dependent decrease in torque for older adults; however, this might simply be due to their decreased range of motion [2]. Other studies have found no *Tθ* changes for DF [3] or PF [4]. Studies on the *Tω* relation have generally shown reduced torque capability for concentric velocities for older DF [2], [5] and PF [5], [6] muscles, but some have found that eccentric torque production is preserved in older adults [7], [8]. Technical aspects of *Tω* determination may play a role in variations between studies. Many studies select the peak torque values generated at a range of joint angular velocities, and scale the data to the maximal value of the isometric *Tθ* curve [5], [9]. However, these peak torque data points may occur at different joint angles, therefore appropriate adjustments should be made to account for the shape of the underlying *Tθ* relation [10].

Although the *Tθ* and *Tω* relations reflect the force-length [11] and force-velocity [12] relations of human muscle, the exact relation between joint and muscle properties also depends on the stiffness of the muscular series elastic elements, which are characterized by the *TΔL* relation. Studies on DF stiffness are scarce, while PF stiffness findings are equivocal. Quick-release studies [13], [14], [15] suggest an increase in PF stiffness with age, but an ultrasonography study showed a decrease [16]. This inconsistency may be due to measurement methods; the quick-release technique measures total muscle-tendon stiffness (*K_MT_*, including external tendon, aponeurosis, and within sarcomeres), while the ultrasonography study measured the stiffness of the external tendon *K_ET_*. Animal studies have indicated that *K_ET_* is greater than stiffness of the aponeurosis *K_AP_* ([17], [18], [19]; although see [20], [21]), so greater *K_MT_* in older adults could result from an increase in *K_AP_*, which can be measured in humans using ultrasonography.

It is important to recognize that the joint *Tθ*, *Tω*, and *TΔL* relations are closely intertwined, and that age-related changes in series elasticity may occur in tandem with *Tθ* and *Tω* modifications, thereby complicating interpretations based on *Tθ* and/or *Tω* measurements alone. The *Tθ* relation affects the shape of the *Tω* relation [22], [23], and series elastic stiffness (*TΔL*) influences muscle fiber length and velocity, altering the shapes of the *Tθ* and *Tω* relations [24]. Therefore, measuring *Tθ* and *Tω* characteristics in parallel with the *TΔL* relation may offer additional insight into age-related differences in functional capability.

Accordingly, the purpose of this study was to investigate age-related differences in static (*Tθ*), dynamic (*Tω*), and elastic (*TΔL*) characteristics of both dorsi- (DF) and plantarflexor (PF) muscles controlling the ankle joint in young and older adults. Based on evidence from previous studies, we anticipated that active community dwelling older adults would have smaller isometric torque capacities, slower concentric muscular properties (i.e. less torque at a given velocity on the *Tω* relation), and stiffer series elasticity in the aponeurosis *TΔL* relation when compared to active young adults. We also expected to find greater age-related differences for the PF muscle group than in the DF, based on more atrophy of Type II fibers in the gastrocnemius [25]. Measuring all three *Tθ*, *Tω*, and *TΔL* properties in the DF and PF antagonist muscle groups provided a comprehensive snapshot of age-related differences in ankle joint function.

## Methods

Twelve young and twelve older independent community-dwelling adults without musculoskeletal or neurological impairments participated in the study (balanced for gender, Table 1). Physician's clearance was obtained for all older subjects. Prior to participating, subjects read and signed an informed consent document approved by the University of Massachusetts Amherst Institutional Review Board. Subjects performed two experimental protocols, with all measurements taken on the left leg.

**Table 1:** Subject characteristics.

Subject Group	N	Age (yrs)		Height (m)		Mass (kg)	
		Mean±SD	Range	Mean±SD	Range	Mean±SD	Range
Young Male	6	27±3	21–30	1.81±0.06	1.70–1.85	76.9±8.2	68.3–87.5
Young Female	6	26±3	21–31	1.65±0.08	1.52–1.74	57.2±6.6	49.9–65.8
Older Male	6	73±5	67–79	1.77±0.08	1.68–1.88	91.7±10.3	74.0–102
Older Female	6	70±5	66–78	1.66±0.09	1.60–1.70	72.6 ±17.0	59.3–77.4

N =  number of subjects; SD =  between-subjects standard deviation.

### Torque-Angle (*Tθ*) and Torque-Angular Velocity (*Tω*) Measurements

#### Experimental Setup

To measure the *Tθ* and *Tω* relations, a series of isometric and isovelocity muscular efforts were performed on a Biodex System 3 dynamometer [26], [27] (Biodex Medical Systems, Shirley, NY). Ankle torque and angular displacement were sampled from the dynamometer at 1000 Hz and 16-bit resolution with software written in Visual Basic 6.0 (Microsoft Corporation, Redmond, WA).

#### Protocol

Subjects sat upright with their trunk inclined backwards 45° from the vertical and arms crossed in front of their chest. After sub-maximal warm-up efforts, passive joint torque was measured by having the dynamometer slowly (20°/s) move the ankle joint through its range of motion without active subject resistance. Isometric (*Tθ*) and isovelocity (*Tω*) dynamometer trials were done on separate days to minimize fatigue. Two trials were performed at each joint angle (*θ*) and angular velocity (*ω*), with trial order randomized and a one-minute rest between trials. The knee remained fixed at 100° for DF and 90° for PF. For isometric trials the ankle position was varied at five different angles evenly spaced throughout each subject's full range of motion. Concentric isovelocity trials were performed at 20°/s, and from 30 to 240°/s in 30°/s increments. Eccentric trials were performed at −150, −60, and −30°/s. In all trials, passive elastic, gravitational, and inertial torque contributions were assessed to calculate the active muscular torque [28]. The passive torque-angle data were averaged across dorsi/plantarflexion trials (typically three per subject); a third-order polynomial was fit to these mean data to generate a passive torque-angle relationship. In the active trials, the polynomial was evaluated at the each instantaneous joint angle and subtracted from the measured torque. Torque contributions due to the weight and inertia of the foot, estimated from de Leva [29], and dynamometer arm, measured experimentally, were also subtracted. We did not account for joint viscosity and friction, as these make negligible contributions to the net joint torque compared with passive elastic, gravitational, and inertial effects [30].

#### Data Processing – Torque Angle (Tθ)

For each subject, the larger of the two maximal isometric joint torque measurements at each ankle angle (*T_IM_*) was used to construct a *Tθ* relationship. The *Tθ* relationship for each subject was expressed as a second order polynomial fit to the DF and PF *T_IM_* vs. *θ* data using a least squares approach. The maximum of the polynomial within the measured range of motion was used to define the maximum isometric torque *T*_0_ and the optimal angle *θ*_0_.

### Data Processing – Torque Angular Velocity (Tω)

**Figure 1:**
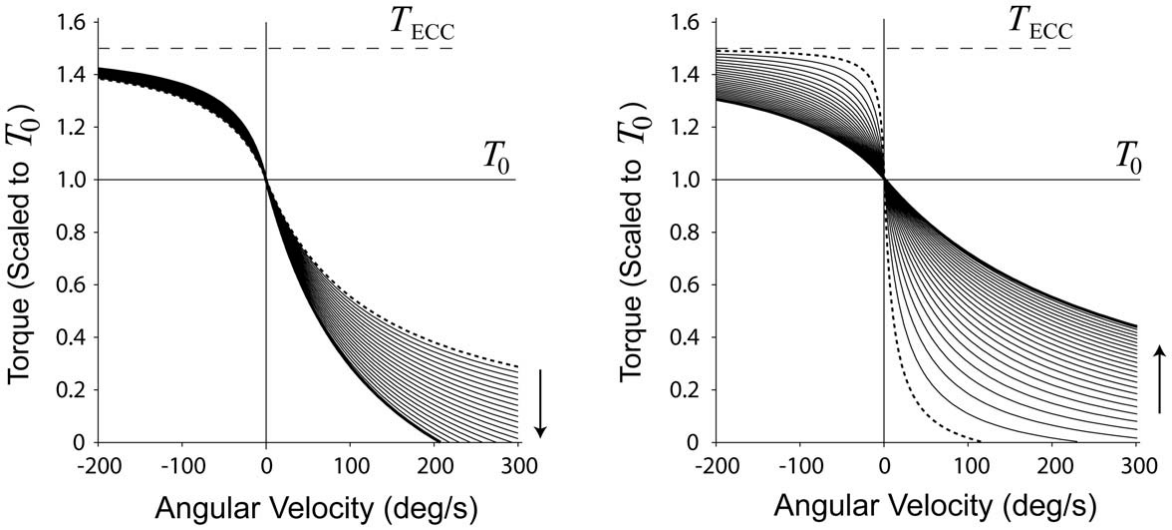
Effects of changing the coefficients describing the torque-angular velocity relation (*Tω*). Left: effect of varying *A_Tω_* from 0.02 to 0.62 while keeping *B_Tω_* =  2.25. Right: effect of varying *B_Tω_* from 0.2 to 5.0 while keeping *A_Tω_* = 0.1. The eccentric plateau (*T_ECC_*) was set to 1.5 *T*_0_. Dashed and thick solid lines indicate small and large values for each coefficient, respectively.

The peak torque *T_IV_* and corresponding *ω* from each isovelocity trial were used to construct a *Tω* relation. At peak torque all shortening/lengthening occurs via the contractile component, i.e. elastic component velocity is momentarily zero. Measured *Tω* data were also adjusted to account for *Tθ* effects [2], [10], [31]. For each subject, the relation between *θ* and *ω* coinciding with the *T_IV_* data points was established using linear regression, and used with the *Tθ* relation to predict the isometric torque capability corresponding to each *T_IV_* value. Each original *Tω* data point was divided by its angle-specific isometric torque capability to give scaled isovelocity peak torque (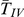.) values (if 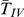.* = 1* then *T_IV_* was equal to the isometric torque at that angle). A rectangular hyperbola was fit to these scaled *Tω* data. Based on Hill [32], if *ω* (rad/s) is positive (concentric), then
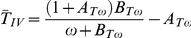
where *A_Tω_* and *B_Tω_* are coefficients describing the shape of the scaled *Tω* relation (Figure 1). For eccentric conditions, when *ω* is negative, based on FitzHugh [33]

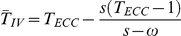
where 
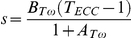
and *T_ECC_* defines the eccentric plateau.

### Torque-Extension (*TΔL*) Measurements

#### Experimental Setup

**Figure 2:**
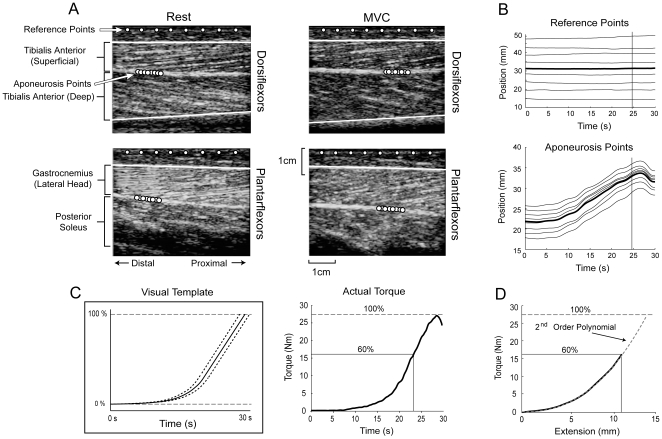
Methodology for measuring musculotendon series elasticity. A: Ultrasound stills from the start (left) and end (right, at MVC) of a dorsi- (top) and plantarflexion (bottom) ramped trial. The white dots indicate points of interest, including a set of reference points near the skin, and a set of points on the aponeurosis of each muscle. The motion of the points was tracked using an automated cross-correlation algorithm. B: Example of the horizontal displacements of the reference points (top) and aponeurosis points for a dorsiflexion trial (thick line = average). C: The visual torque-time template (left) and the actual dorsiflexion torque produced (right). D: The resulting torque vs. extension plot. A second order polynomial was fit to the torque up to 60% MVC, and then extended up to MVC (dashed line).

To estimate DF and PF *TΔL* relationships, ankle torque was measured as subjects performed a series of ramped maximal isometric efforts on the dynamometer. The tibialis anterior, lateral head of the gastrocnemius, and soleus muscles were imaged using an Acuson 128XP real-time ultrasonic scanner with a linear-array probe (7.5 MHz, 50 mm scanning length, B-mode). The ultrasound probe was orientated along the mid-sagittal axis of each muscle, with transmission gel used for improved acoustic coupling [34], [35]. Ultrasound images (Figure 2A) were sampled at 30 Hz and saved to magnetic tape. With the knee extended, the ankle was positioned at 90° where passive contributions are minimal [36]. The torque data were sampled at 900 Hz with 16-bit resolution, using a 5V analog pulse for synchronization with the ultrasound data.

#### Protocol

Subjects performed two blocks of trials (DF, PF), with block order balanced across subjects. Within each block, two maximal voluntary contractions (MVCs) were performed to establish maximal isometric ankle torque, followed by five trials with torque and ultrasound measurements. In each 30-second trial, subjects were asked to match their ankle torque to a predefined green target force template that increased exponentially from 0 to 30% MVC, and linearly from 30 to 100% MVC (Figure 2C, solid line). The initial exponential increase was to ensure a slow rate of extension to facilitate subsequent image analysis. Red lines defined acceptable torque variation (Figure 2C, broken lines). During each trial, the subject's actual ankle torque appeared in real time. Subjects were instructed to follow the green line as closely as possible. Trials were separated by two-minute rest periods.

#### Video Capture and Preprocessing

The ultrasound video was parsed into individual trials and converted to digital format (AVI, 720×480 pixels) using a video capture system (Studio MovieBox USB, Pinnacle Systems). Subsequent processing was done using Matlab™ (Version 7, The MathWorks, Natick MA). The 900 Hz torque data were downsampled to 30 Hz and synchronized with the ultrasound video using the rising edge of the analog pulse.

#### Tracking of Aponeurosis Extension

To measure extension, multiple points on the ultrasound images were identified: one set of eight superficial reference points evenly spaced near the skin, and a cluster of eight points along the aponeurosis (see Figure 2A). The movement of all points was automatically tracked using a two-dimensional cross-correlation tracking algorithm [37]. The tibialis anterior aponeurosis extension was assumed to represent the DF muscles (including the tibialis anterior, extensor hallucis longus, extensor digitorum longus, and peroneus tertius), while the extension of the combined gastrocnemuis and soleus aponeuroses represented the PF muscles.

#### Data Processing

Horizontal and vertical point displacements and the torque data were smoothed using a Butterworth digital filter with optimal cut-off frequencies determined through residual and power spectral analyses [38]. The multiple reference and aponeurosis point displacements were averaged to give a single reference and aponeurosis time-series (Figure 2B). After subtraction of reference point motion to account for possible probe movement relative to the skin, the magnitude of the aponeurosis displacement vector was expressed as extension (*ΔL*) from the rest position. Highly variable data above 60% MVC torque were excluded from the subsequent fitting of a second order polynomial [16], [39], [40] to the ankle torque (*T*) vs. *ΔL* data (Figure 2D):
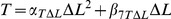
where *α_TΔL_* and *β_TΔL_* are shape coefficients. The linear stiffness (*K*) was calculated as the slope of the fitted polynomials (i.e. 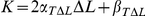.) at three different torque levels (*K_Low_* = 5 Nm [DF], 15 Nm [PF]; *K_Med_* = 15 Nm [DF], 30 Nm [PF]; *K_High_* = 30 Nm [DF], 60 Nm [PF]). The maximum extension of the series elastic components (*ΔL_MAX_*) was assessed by evaluating the polynomial equation at MVC.

### Statistics

Due to technical issues affecting torque measurement, one of the young male subjects was excluded from the *Tθ* and *Tω* analysis. Separate three-way ANOVAs (age [Y,O] x gender [M,F] x muscle [PF,DF]) were performed on each dependent variable. The dependent variables included two *Tθ* variables (*T*_0_, *θ*_0_) and three *Tω* variables (*A_Tω_*, *B_Tω_*, and *T_ECC_*). For the *TΔL* relations, the three dependent variables were *α_TΔL_*_,_ *β_TΔL_* and *ΔL_MAX_*. Separate two-way ANOVAs (age x gender) were performed for DF and PF linear stiffness measures (*K_Low_*, *K_Med_*, *K_High_*); an additional one-way ANOVA was performed to test for muscle group difference at the same absolute torque level (15 Nm). Outliers were defined as data points that exceeded the mean by more than three times the inter-quartile range. Statistical significance for all tests was set at *p≤*.05.

## Results

### Torque-Angle (*Tθ*)

**Figure 3:**
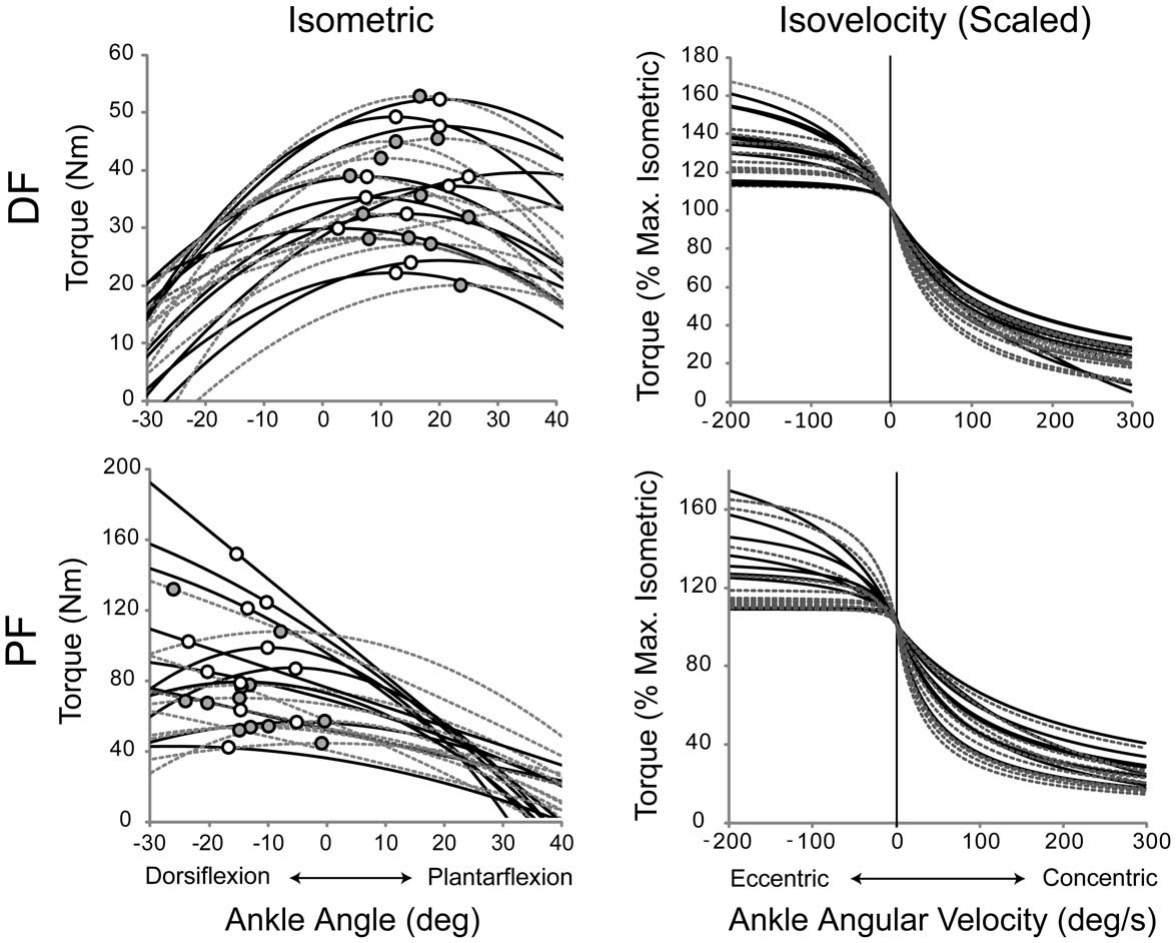
Equations representing the best fit between the experimental data and second order polynomials (torque-angle relation; isometric) and rectangular hyperbolas (torque-angular velocity relation; isovelocity) for young (solid black lines) and older (dashed gray lines). The solid circles positioned on the isometric curves represent the peak isometric torque. For some subjects, the peak did not occur within the subject's range of motion, in these cases the solid circle is positioned at the end of the range of motion. The isovelocity fits are scaled to the peak isometric torques.

**Figure 4:**
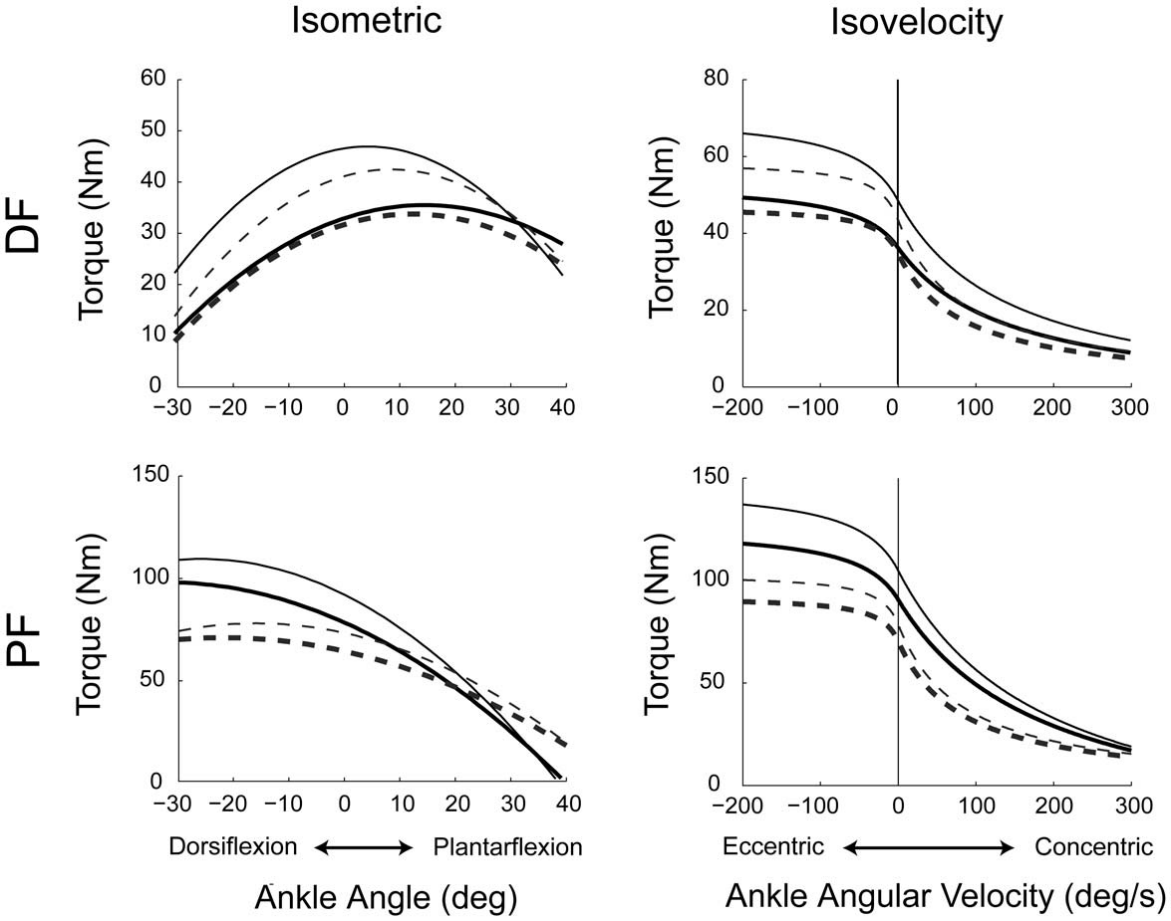
Average young (black solid lines) and older (gray dashed lines) torque-angle (left) and torque-angular velocity (right) curves. Both measured (thick lines) and co-activation adjusted (thin lines; see Discussion) data fits are shown.

**Figure 5:**
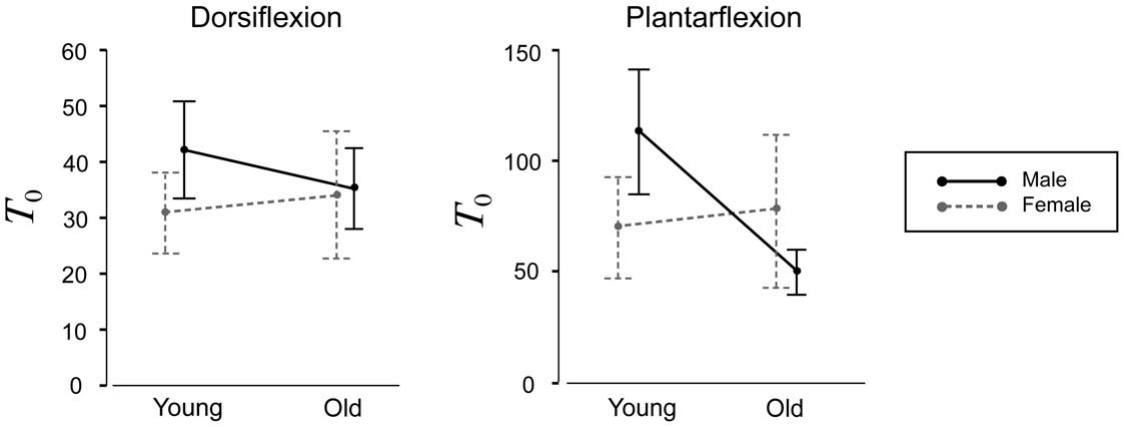
Interaction plots for maximal isometric torque (*T*_0_).

Individual subject *Tθ* and *Tω* relations are shown in Figure 3; summary statistics are given in Table 2, with age group mean curves shown in Figure 4. For maximal isometric torque *T*_0_, there was a main effect of age, with *T*_0_ greater in the young compared to old (*p* = .028), and a main effect of muscle, with PF greater then DF (*p*<.001). There was also a three-way interaction between age, gender, and muscle (*p* = .034) (Figure 5). No age-related differences were found in females for DF *T*_0_ (*p* = .794) or PF *T*_0_ (*p* = .161), or for DF *T*_0_ in males (*p* = .587), but older males had significantly weaker PF compared to younger males (*p*<.001). There was a main effect of muscle for optimal angle *θ*_0_ (*p*<.001), with *θ*_0_ occurring at a more plantarflexed position for DF (at +14° of plantarflexion) than for PF (at −14° of dorsiflexion), but there were no *θ*_0_ effects for age (*p* = .834) or gender (*p* = .201).

**Table 2:** Parameters characterizing torque-angle (*Tθ*) and torque-angular velocity (*Tω*) data (mean±between-subjects standard deviation).

Group	Muscle Group	*Tθ*		*Tω*		
		*T*_0_ (Nm)	*θ*_0_ (°)	*A_Tω_*	*B_Tω_*	*T_ECC_*
Young Male a	DF	42.1±8.70	14.3±5.28	0.030±0.078	2.12±0.43	1.48±0.31
	PF	114±28.1	−12.7±2.48	0.350±0.585	3.19±3.04	1.37±0.35
Young Female	DF	30.9±7.40	13.6±8.25	0.241±0.358	2.81±1.27	1.43±0.20
	PF	70.9±22.8	−14.3±7.65	0.240±0.535	2.15±1.58	1.35±0.41
Young Average	DF	36.5±8.10	13.9±6.77	0.136±0.218	2.47±0.85	1.46±0.26
	PF	92.5±25.5	−13.5±5.07	0.295±0.560	2.67±2.31	1.36±0.38
Older Male	DF	35.2±7.20	16.0±6.15	0.029±0.044	1.31±0.28	1.30±0.12
	PF	61.4±9.72	−12.8±7.74	0.034±0.068	1.53±0.82	1.21±0.20
Older Female	DF	34.1±11.5	12.4±6.74	0.008±0.014	1.59±0.29	1.39±0.27
	PF	78.5±34.3	−13.8±8.94	0.050±0.123	1.41±1.05	1.35±0.33
Older Average	DF	34.7±9.35	14.2±6.45	0.019±0.029	1.45±0.29	1.35±0.20
	PF	69.9±22.0	−13.3±8.34	0.042±0.096	1.47±0.94	1.28±0.27
Main Effects^b^		A, M	M	-	A	-
Interactions^b^		A X G X M	-	-	G X M	-

a One outlier is excluded.

b Significant main effects and interactions for age (A), gender (G), and muscle (M).

DF, PF: dorsiflexors, plantarflexors.

*T*_0_: maximal isometric joint torque.

*θ*_0_: ankle angle at *T*_0_ (DF =  Negative; PF =  Positive).

*A_Tω_*, *B_Tω_*: shape coefficients for *Tω* relation; units for *B_Tω_* are rad/s.

*T_ECC_*: eccentric plateau of *Tω* relation (relative to *T*_0_).

### Torque-Angular Velocity (*Tω*)

Compared to the younger individuals, older adults produced less torque relative to *T*_0_ for concentric velocities, as indicated by the smaller *Tω* shape coefficient *B_Tω_* (Table 2 and Figures 3 and 4; age main effect *p* = .003). There were no main effects of gender (*p* = .935) or muscle (*p*  = .977). There were no effects of age, gender, or muscle on the *Tω* parameters *A_T_*_ω_ (*p*>.110 for all tests) or *T_ECC_* (*p*>.192 for all tests).

### Torque-Extension (*TΔL*)

**Figure 6:**
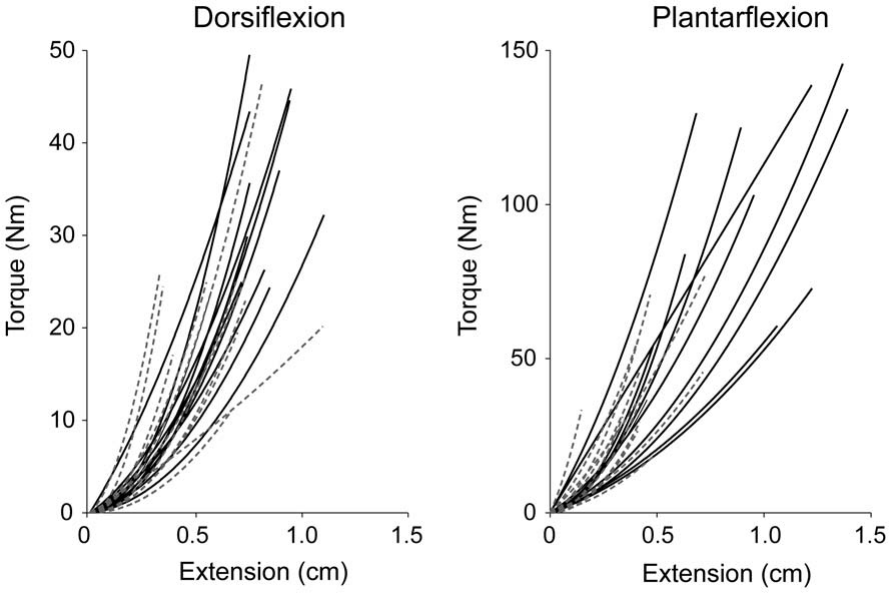
Second order polynomial fits to the young (solid black lines) and older (broken gray lines) torque-extension data from the ultrasound experiments.

Individual subject *TΔL* relations are shown in Figure 6. For these second order polynomial relations, older subjects had larger *α_TΔL_* coefficients (Table 3; *p* = .009), but no differences for *β_TΔL_* (*p* = .457). Both coefficients were larger for PF compared to DF (*α_TΔL_*; *p* = .002; *β_TΔL_*; *p*<.001). There were no gender differences for either coefficient (*α_TΔL_*; *p* = .706; *β_TΔL_*; *p*<.343). There were no age effects for DF at any linear stiffness level (*K_Low_*, *K_Med_*, *K_High_*) (p>.691), and none for PF at *K_Low_* (*p*  = .143) and *K_Med_* (p = .058). However, *K_High_* was greater for the older subjects (*p*  = .031). There were no gender differences at any *K* for DF (*p*>.538) or PF (*p*>.231). When comparing *K* at the same absolute torque level (15 Nm), the PF muscles were stiffer than the DF (*p*<.001). Older adults had smaller maximal aponeurosis extensions (5.7 mm) than the younger subjects (8.8 mm) (*ΔL_MAX_*; *p*<.001). There was an age by muscle interaction for maximal extension (*p* = .015), with PF *ΔL_MAX_* smaller than DF (*p* = .017) for the older subjects, but not for the young (*p* = .388).

**Table 3:** Parameters describing the torque-extension (*TΔL*) data (mean±between-subjects standard deviation).

Group	Mus.	*ΔL_MAX_* (mm)	*α_TΔL_*	*β_TΔL_*	*K_Low_*	*K_Med_*	*K_High_*
Young Male	DF	8.4±1.0	0.491±0.221	0.570±0.643	3.51±0.64	5.62±0.92	7.75±1.42
	PF	11±3.5	0.835±0.697	4.22±3.55	8.61±2.25	11.1±2.75	14.6±4.31
Young Female	DF	8.0±1.8	0.386±0.194	0.577±0.550	2.83±0.59	4.77±1.09	6.69±1.57
	PF	7.8±3.0	0.809±0.733	4.64±4.17	8.59±3.61	10.8±4.48	14.1±6.22
Young Average	DF	8.2±1.4	0.439±0.208	0.574±0.597	3.17±0.62	5.20±1.01	7.22±1.50
	PF	9.4±3.3	0.822±0.715	4.43±3.86	8.60±2.93	11.0±3.62	14.4±5.27
Older Male^a^	DF	8.5±4.7	0.578±0.617	0.893±0.816	3.22±1.75	5.34±3.02	7.45±4.28
	PF	3.5±1.4	2.10±5.11	1.42±1.35	11.3±1.41	15.9±1.96	28.5±15.2
Older Female	DF	6.0±1.8	0.809±0.678	0.197±0.403	3.81±1.48	6.57±2.59	9.27±3.67
	PF	4.7±2.0	1.64±1.73	5.52±5.59	8.55±2.67	11.9±3.40	19.1±10.1
Older Average	DF	7.3±3.3	0.694±0.648	0.545±0.610	3.52±1.62	5.96±2.81	8.36±3.98
	PF	4.1±1.7	1.87±3.42	3.47±3.47	9.93±2.04	13.9±2.68	23.8±12.7
Main Effects^b^		A	A, M	M	-	M	A (PF)
Interactions^b^		A×M	-	-	-	-	-

a One outlier is excluded.

b Significant main effects and interactions for age (A) and muscle (M).

DF, PF: dorsiflexors, plantarflexors.

*ΔL_MAX_*: maximum extension of aponeurosis.

*α_TΔL_*, *β_TΔL_*: shape coefficients for the *TΔL* relation.

*K*: Linear stiffness coefficients at different absolute torque levels; for DF: [*K_Low_*, *K_Med_*, *K_High_*] = [5, 15, 30 Nm]; For PF: [*K_Low_*, *K_Med_*, *K_High_*] = [15, 30, 60 Nm]. Stiffness units are Nm/mm.

## Discussion

The hypothesis of lower maximal isometric torques (*T*_0_) in the older adults was supported partially; only the older male subjects had lower *T*_0_, and only in the PF muscle group. There was stronger support for the hypotheses of decreased concentric force capabilities and increased series elastic stiffness in the older adults; however, the latter was observed only for PF. We also expected to find greater age-related differences for PF compared to DF due to previous reports of Type II fiber atrophy in the gastrocnemius in older adults. This was observed for the male PF *T*_0_ as well as the PF stiffness; however, age-related reductions in concentric force capabilities were found for both DF and PF. No differences were found between age groups in the optimal joint angle of the *Tθ* relation, or in eccentric torque capabilities.

### Torque-Angle

As in prior studies [1], [2], [41], we found age-related deficits in maximal isometric joint strength (*T*_0_), but only for PF in male subjects; females showed no age-related differences. Kent-Braun and Ng [42] reported a similar interaction for DF, with *T*_0_ greater in young men than older, but not in the females. Other support for an age and gender interaction is provided by Winegard et al. [4], in which older female subjects had larger PF *T*_0_ values than older males, and did not show significant age-related deficits in strength. These findings could be related to age-related decreases in male testosterone concentrations [43], which is associated with decreased muscle mass and strength [44], [45]. We saw no age-related differences for the angle of peak torque (*θ*_0_), consistent with Simoneau and colleagues [46], who found no variations with age in the DF and PF *Tθ* relations. We also observed that the angle at maximum torque (*θ*_0_) was not different between the age/gender groups. Relative to neutral (0°), both DF *θ*_0_ (+14° of plantarflexion) and PF *θ*_0_ (−14° of dorsiflexion) were similar to other studies [2], [22], [47], [48], [49], [50].

Our estimates of *T*_0_ were based on a second order polynomial that represented the *Tθ* relation. Average DF *T*_0_ values were ∼37 Nm for the younger subjects and ∼35 Nm for the older subjects. These data agree with literature values that range from ∼22 to 44 Nm for both young and older adults [1], [2], [5], [46], [51], [52]. For PF *T*_0_, values averaged 93 Nm for young and 70 Nm for older males, while literature values range from 104 to 232 Nm for young males and from 100 to 125 Nm for older males [1], [4], [5], [46], [51], [53]. Our relatively low results could be due to several factors, including the use of a knee angle of 90°, at which the gastrocnemius is shorter than optimum length [50], reducing the net plantarflexor torque. We also report active torque after subtracting for passive torque contributions, and were stringent concerning proper joint alignment and/or upper body fixation during the experimental trials [54].

### Torque-Angular Velocity

The *Tω* relation was characterized by the shape parameters *A_Tω_* and *B_Tω_,* akin to the dynamic constants of the Hill force-velocity equation [12] and independent of *T*_0_. Figure 1 illustrates that independently increasing *A_Tω_* produces lower torque at any given concentric velocity, while increasing *B_Tω_* leads to greater concentric, but decreased eccentric force capability.

The shape of the *Tω* relation differed between age groups, with weaker/slower concentric force capabilities (lower *B_Tω_*) in older DF and PF, but without a concomitant deficit in eccentric strength as indicated by the eccentric torque plateau (*T_ECC_*). These data are consistent with other reports that concentric capability is reduced with aging [2], [5], [6], [55], but that eccentric torque production is preserved [7], [8], [9], [56], [57], [58]. Because we accounted for individual subject differences in strength, muscle moment arms, and muscle torque-angle relations, our *Tω* findings represent a difference in the intrinsic force-velocity properties of the ankle muscles. This may be related to an age-related decrease in the size and/or number of fast-twitch Type II muscle fibers in the dorsiflexor tibialis anterior and the plantarflexor gastrocnemius [59]. With aging the percentage of Type IIa fibers decreases by about 9% in the tibialis anterior [60]. Although gastrocnemius Type IIa and Type IIb fiber number remains stable with increased age, these fibers shrink by about 13–31% in comparison with sedentary young adults [25].

### Series Elasticity

The change in series elastic component length as a function of joint torque (i.e. the *TΔL* relation) was represented by a second order polynomial with shape coefficients *α_TΔL_* and *β_TΔL_*. The squared coefficient *α_TΔL_* has a large influence on the rate of torque increase with increasing extension, and this coefficient was larger for the older subjects, indicating greater non-linearity in the *TΔL* relation. There were no age differences in the *β_TΔL_* coefficient, which has a smaller role in determining the shape of the *TΔL* relation. To determine linear stiffness, we computed the slope of the non-linear *TΔL* relation at three different relative torque levels (*K_Low_, K_Med_, K_High_*). Although we found no age differences among DF linear stiffness values at any torque level, older PF muscles had greater stiffness when evaluated at the high torque level (*K_High_*). Greater PF stiffness in older adults agrees with other studies [13], [14], [61], and may offset the age-related decrement in velocity-dependent force capabilities, as force can rise faster if the contractile component is attached to a stiffer series elastic component rather than a more compliant one [62].

This study also found *TΔL* differences between the muscle groups. Compared to DF, both *α_TΔL_* and *β_TΔL_* polynomial coefficients and the linear stiffness measure at 15 Nm were greater for PF. This is supported by literature reports of aponeurosis stiffness and Young's modulus (*E*) (at maximal torque) *in vivo*, with lower values (stiffness = 32 N/mm; *E* = 0.563 Gpa) reported for DF [63] than for the PF muscles (stiffness = 467 N/mm; *E* = 1.474 GPa)[39]. We did not compute *E*, since it requires explicit measures of series elastic component length and cross-sectional area [64]. Our findings of increased PF stiffness are also supported by evidence that muscle elastic characteristics are influenced by physiological function [65]; PF would be expected to have a higher stiffness to support much higher static and dynamic loads compared to DF [66]. This is one of the first reports on DF stiffness in older adults, as most studies focus on PF because they are purported to make a larger contribution to activities of daily living [67]. On the other hand, DF can generate significant torque and contribute to ankle joint stiffness that is important for postural stabilization [68].

It is important to note the limitations associated with these interpretations of series-elastic stiffness. Although we only measured aponeurosis extension in the longitudinal direction, the aponeurosis stretches bi-axially in the longitudinal and transverse planes [20], [69]. This complex behavior results in a variable aponeurosis stiffness [70], which we characterized with a second-order polynomial, and made linear approximations of the stiffness (*K*) at different torque values to facilitate group comparisons. Although we assumed that the aponeurosis acts in series with the muscle fibers and external tendon, the force expressed across these structures may differ due to the complex geometry of the musculotendon unit [71]. We did not normalize the stiffness measures by the cross-sectional area and rest length of the elastic structures. Additionally, the gastrocnemius and soleus aponeuroses are separate structures linked by transverse collagen fibers, which can move independently during PF torque production, i.e. there is inter-aponeurosis shear [72]. Our ultrasound machine had insufficient resolution to make this subtle distinction. Thus, our PF aponeurosis extension values represent an average for the gastrocnemius and soleus (the tracking points spanned both aponeuroses).

### Antagonist Co-Activation

In measuring resultant joint torque to assess muscle function, we made the common assumption of no antagonist co-activation [2], [5], [14], [22], [51]. This assumption is likely false in maximal joint torque efforts, and could cause agonist torque capabilities to be underestimated [73], which in turn would lead to inaccurate muscular properties. To assess possible influences of antagonistic co-activation, we estimated the percentage of antagonistic co-activation (*%CoAct*) from the isometric data of Simoneau et al. [41]

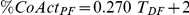


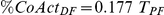
where *T_DF_* and *T_PF_* are the agonist DF and PF torques (Nm). For each muscle group the antagonistic torque contribution (*T^Antag^,* Nm) was computed as
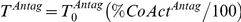



We found that including antagonistic contributions increased agonist DF *T_0_* by 35% in young and 26% in older subjects, and increased agonist PF *T*_0_ by 16% in young and 12% in older subjects. The most pronounced change was for isometric DF, in which the small differences between the young and old subjects became much greater with the inclusion of antagonism (Figure 4). This was because the younger subjects had stronger PF muscles and therefore greater antagonistic torque during the isometric DF trials.

Including antagonistic contributions may also influence stiffness measurements. Agonist muscles produce more torque than the dynamometer measures when antagonist muscles produce opposing torques. Magnusson et al. [39] demonstrated that antagonistic influences are small but significant for PF in male adults (average age: 37 yrs), such that Achilles tendon force was underestimated by ∼2.6% when antagonist coactivation was ignored. Lower torques for a given tendinous extension would cause stiffness to be underestimated. However, in the present study the degree of underestimation would be smaller for the older subjects and the DF muscles, since in both cases the magnitudes of the joint torques were smaller than those measured by Magnusson and colleagues.

Our simple antagonism model assumed that antagonist co-activation is dictated by agonist torque level alone, is independent of joint angular velocity [74], [75], and that the torque/co-activation relation was the same for both age groups [41]. However, the degree of antagonistic co-contraction is likely task-specific, and may be influenced by joint angle [46], [76], angular velocity [77], and whether the muscular effort is isometric, concentric, or eccentric [78], [79]. One possibility is to use electromyography to estimate co-activation [80], but some have questioned the reliability of antagonist torque estimated in this manner [76]. Therefore, the exact magnitude of the co-activation adjusted results should be viewed with caution, but the simple model used here demonstrates the importance of including antagonism when predicting muscular properties.

### Conclusions

Many clinical studies use maximal isometric strength as a marker of functional ability [81], [82]. However, the present study has shown additional age-related differences in the dynamic properties of the ankle muscles, with slower concentric force capabilities and stiffer series elasticity in the older adults. This assessment of static, dynamic, and elastic joint properties affords a comprehensive view of age-related modifications in muscle function. Future work should further investigate links between age-related differences in these properties, which together may represent adaptations to physiological and lifestyle changes in older adults. Since the subjects in the present study were all healthy and active members of the community, we speculate that these differences represent a normal aging process.

## References

[1] Vandervoort AA, McComas AJ (1986). Contractile changes in opposing muscles of the human ankle joint with aging.. J Appl Physiol.

[2] Lanza IR, Towse TF, Caldwell GE, Wigmore DM, Kent-Braun JA (2003). Effects of age on human muscle torque, velocity, and power in two muscle groups.. J Appl Physiol.

[3] van Schaik CS, Hicks AL, McCartney N (1994). An evaluation of the length-tension relationship in elderly human ankle dorsiflexors.. J Gerontol.

[4] Winegard KJ, Hicks AL, Vandervoort AA (1997). An evaluation of the length-tension relationship in elderly human plantarflexor muscles.. J Gerontol A Biol Sci Med Sci.

[5] Thelen DG, Schultz AB, Alexander NB, Ashton-Miller JA (1996). Effects of age on rapid ankle torque development.. J Gerontol A Biol Sci Med Sci.

[6] Thom JM, Morse CI, Birch KM, Narici MV (2007). Influence of muscle architecture on the torque and power-velocity characteristics of young and elderly men.. Eur J Appl Physiol.

[7] Porter MM, Myint A, Kramer JF, Vandervoort AA (1995). Concentric and eccentric knee extension strength in older and younger men and women.. Can J Appl Physiol.

[8] Porter MM, Vandervoort AA, Kramer JF (1997). Eccentric peak torque of the plantar and dorsiflexors is maintained in older women.. J Gerontol A Biol Sci Med Sci.

[9] Klass M, Baudry S, Duchateau J (2005). Aging does not affect voluntary activation of the ankle dorsiflexors during isometric, concentric, and eccentric contractions.. J Appl Physiol.

[10] Caldwell GE, Adams WB, Whetstone MR (1993). Torque/velocity properties of human knee muscles: peak and angle-specific estimates.. Can J Appl Physiol.

[11] Gordon AM, Huxley AF, Julian FJ (1966). The variation in isometric tension with sarcomere length in vertebrate muscle fibres.. J Physiol.

[12] Hill AV (1938). The heat of shortening and the dynamic constants of muscle.. Proc R Soc Lond B Biol Sci.

[13] Ochala J, Lambertz D, Van Hoecke J, Pousson M (2005). Effect of strength training on musculotendinous stiffness in elderly individuals.. Eur J Appl Physiol.

[14] Ochala J, Lambertz D, Pousson M, Goubel F, Hoecke JV (2004). Changes in mechanical properties of human plantar flexor muscles in ageing.. Exp Gerontol.

[15] Blanpied P, Smidt GL (1993). The difference in stiffness of the active plantarflexors between young and elderly human females.. J Gerontol.

[16] Onambele GL, Narici MV, Maganaris CN (2006). Calf muscle-tendon properties and postural balance in old age.. J Appl Physiol.

[17] Lieber RL, Leonard ME, Brown CG, Trestik CL (1991). Frog semitendinosis tendon load-strain and stress-strain properties during passive loading.. Am J Physiol.

[18] Delgado-Lezama R, Raya JG, Munoz-Martinez EJ (1997). Methods to find aponeurosis and tendon stiffness and the onset of muscle contraction.. J Neurosci Methods.

[19] Ettema GJ, Huijing PA (1989). Properties of the tendinous structures and series elastic component of EDL muscle-tendon complex of the rat.. J Biomech.

[20] Scott S, Loeb G (1995). Mechanical properties of aponeurosis and tendon of the cat soleus muscle during whole muscle isometric contractions.. J Morphol.

[21] Muramatsu T, Muraoka T, Takeshita D, Kawakami Y, Hirano Y (2001). Mechanical properties of tendon and aponeurosis of human gastrocnemius muscle in vivo.. J Appl Physiol.

[22] Bobbert MF, van Ingen Schenau GJ (1990). Isokinetic plantar flexion: experimental results and model calculations.. J Biomech.

[23] Taylor NA, Cotter JD, Stanley SN, Marshall RN (1991). Functional torque-velocity and power-velocity characteristics of elite athletes.. Eur J Appl Physiol Occup Physiol.

[24] Kawakami Y, Kubo K, Kanehisa H, Fukunaga T (2002). Effect of series elasticity on isokinetic torque-angle relationship in humans.. Eur J Appl Physiol.

[25] Coggan A, Spina R, King D, Rogers M, Brown M (1992). Histochemical and enzymatic comparison of the gastrocnemius muscle of young and elderly men and women.. J Gerontol.

[26] Drouin JM, Valovich-mcLeod TC, Shultz SJ, Gansneder BM, Perrin DH (2004). Reliability and validity of the Biodex system 3 pro isokinetic dynamometer velocity, torque and position measurements.. Eur J Appl Physiol.

[27] Taylor NA, Sanders RH, Howick EI, Stanley SN (1991). Static and dynamic assessment of the Biodex dynamometer.. Eur J Appl Physiol Occup Physiol.

[28] Herzog W (1988). The relation between the resultant moments at a joint and the moments measured by an isokinetic dynamometer.. J Biomech.

[29] De Leva P (1996). Adjustments to Zatsiorsky-Seluyanov's segment inertia parameters.. J Biomech.

[30] Johns R, Wright V (1962). Relative importance of various tissues in joint stiffness.. J Appl Physiol.

[31] Chapman A, Caldwell G, Selbie W (1985). Mechanical output following muscle stretch in forearm supination against inertial loads.. J Appl Physiol.

[32] Hill AV (1970). First and last experiments in muscle mechanics..

[33] FitzHugh R (1977). A model of optimal voluntary muscular control.. J Math Biol.

[34] Maganaris CN, Baltzopoulos V, Sargeant AJ (1998). In vivo measurements of the triceps surae complex architecture in man: implications for muscle function.. J Physiol.

[35] Maganaris CN (2001). Force-length characteristics of in vivo human skeletal muscle.. Acta Physiol Scand.

[36] Siegler S, Moskowitz GD, Freedman W (1984). Passive and active components of the internal moment developed about the ankle joint during human ambulation.. J Biomech.

[37] Loram ID, Maganaris CN, Lakie M (2004). Paradoxical muscle movement in human standing.. J Physiol.

[38] Winter DA (1990). Biomechanics and Motor Control of Human Movement: John Wiley & Sons.

[39] Magnusson SP, Aagaard P, Dyhre-Poulsen P, Kjaer M (2001). Load-displacement properties of the human triceps surae aponeurosis in vivo.. J Physiol.

[40] Magnusson SP, Hansen P, Aagaard P, Brond J, Dyhre-Poulsen P (2003). Differential strain patterns of the human gastrocnemius aponeurosis and free tendon, in vivo.. Acta Physiol Scand.

[41] Simoneau E, Martin A, Van Hoecke J (2005). Muscular performances at the ankle joint in young and elderly men.. J Gerontol A Biol Sci Med Sci.

[42] Kent-Braun JA, Ng AV (1999). Specific strength and voluntary muscle activation in young and elderly women and men.. J Appl Physiol.

[43] Harman SM, Metter EJ, Tobin JD, Pearson J, Blackman MR (2001). Longitudinal effects of aging on serum total and free testosterone levels in healthy men. Baltimore Longitudinal Study of Aging.. J Clin Endocrinol Metab.

[44] Abbasi A, Mattson D, DuthieJr E, Wilson C, Sheldahl L (1998). Predictors of lean body mass and total adipose mass in community-dwelling elderly men and women.. Am J Med Sci.

[45] Baumgartner R, Waters D, Gallagher D, Morley J, Garry P (1999). Predictors of skeletal muscle mass in elderly men and women.. Mech Ageing Dev.

[46] Simoneau E, Martin A, Van Hoecke J (2007). Effects of joint angle and age on ankle dorsi- and plantar-flexor strength.. J Electromyogr Kinesiol.

[47] Belanger AY, McComas AJ, Elder GB (1983). Physiological properties of two antagonist human muscle groups.. Eur J Appl Physiol Occup Physiol.

[48] Ferri A, Scaglioni G, P M, Capodaglio P, Van Hoecke J (2003). Strength and power changes of the human plantarflexors and knee extensors in response to resistance training in old age.. Acta Physiol Scand.

[49] Marsh E, Sale D, McComas AJ, Quinlan J (1981). Influence of joint position on ankle dorsiflexion in humans.. J Appl Physiol.

[50] Sale D, Quinlan J, Marsh E, McComas AJ, Belanger AY (1982). Influence of joint position on ankle plantarflexion in humans.. J Appl Physiol.

[51] Anderson DE, Madigan ML, Nussbaum MA (2007). Maximum voluntary joint torque as a function of joint angle and angular velocity: model development and application to the lower limb.. J Biomech.

[52] Fukunaga T, Ito M, Ichinose Y, Kuno S, Kawakami Y (1996). Tendinous movement of a human muscle during voluntary contractions determined by real-time ultrasonography.. J Appl Physiol.

[53] Karamanidis K, Arampatzis A (2006). Mechanical and morphological properties of human quadriceps femoris and triceps surae muscle-tendon unit in relation to aging and running.. J Biomech.

[54] Oberg B, Bergman T, Tropp H (1987). Testing of isokinetic muscle strength in the ankle.. Med Sci Sports Exerc.

[55] Harries UJ, Bassey EJ (1990). Torque-velocity relationships for the knee extensors in women in their 3rd and 7th decades.. Eur J Appl Physiol Occup Physiol.

[56] Hortobagyi T, Zheng D, Weidner M, Lambert NJ, Westbrook S (1995). The influence of aging on muscle strength and muscle fiber characteristics with special reference to eccentric strength.. J Gerontol A Biol Sci Med Sci.

[57] Poulin MJ, Vandervoort AA, Paterson DH, Kramer JF, Cunningham DA (1992). Eccentric and concentric torques of knee and elbow extension in young and older men.. Can J Sport Sci.

[58] Vandervoort AA, Kramer JF, Wharram ER (1990). Eccentric knee strength of elderly females.. J Gerontol.

[59] Lexell J, Taylor CC, Sjostrom M (1988). What is the cause of the ageing atrophy? Total number, size and proportion of different fiber types studied in whole vastus lateralis muscle from 15- to 83-year-old men.. J Neurol Sci.

[60] Jakobsson F, Borg K, Edström L, Grimby L (1988). Use of motor units in relation to muscle fiber type and size in man.. Muscle Nerve.

[61] Blanpied P, Smidt GL (1993). The difference in stiffness of the active plantar flexors between young and elderly human females.. J Gerontol.

[62] Caldwell GE (1995). Tendon elasticity and relative length: Effects on the Hill two-component muscle model.. J Appl Biomech.

[63] Ito M, Kawakami Y, Ichinose Y, Fukashiro S, Fukunaga T (1998). Nonisometric behavior of fascicle during isometric contractions of a human muscle.. J Appl Physiol.

[64] Maganaris CN, Paul JP (1999). In vivo human tendon mechanical properties.. J Physiol.

[65] Shadwick RE (1990). Elastic energy storage in tendons: mechanical differences related to function and age.. J Appl Physiol.

[66] Roy A, Krebs H, Williams D, Bever C, Forrester L (2009). Robot-aided neurorehabilitation: a novel robot for ankle rehabilitation.. IEEE Trans Robot.

[67] Judge JO, Davis RB, Ounpuu S (1996). Step length reductions in advanced age: the role of ankle and hip kinetics.. J Gerontol A Biol Sci Med Sci.

[68] Winter DA, Patla AE, Prince F, Ishac M, Gielo-Perczak K (1998). Stiffness control of balance in quiet standing.. J Neurophysiol.

[69] Van Donkelaar C, Willems P, Muijtjens A, Drost M (1999). Skeletal muscle transverse strain during isometric contraction at different lengths.. J Biomech.

[70] Azizi E, Roberts T (2009). Biaxial strain and variable stiffness in aponeuroses.. J Physiol.

[71] Epstein M, Wong M, Herzog W (2006). Should tendon and aponeurosis be considered in series?. J Biomech.

[72] Bojsen-Moller J, Hansen P, Aagaard P, Svantesson U, Kjaer M (2004). Differential displacement of the human soleus and medial gastrocnemius aponeuroses during isometric plantar flexor contractions in vivo.. J Appl Physiol.

[73] Simoneau EM, Billot M, Martin A, Van Hoecke J (2009). Antagonist mechanical contribution to resultant maximal torque at the ankle joint in young and older men.. J Electromyogr Kinesiol.

[74] Bazzucchi I, Sbriccoli P, Marzattinocci G, Felici F (2006). Coactivation of the elbow antagonist muscles is not affected by the speed of movement in isokinetic exercise.. Muscle Nerve.

[75] Hubley-Kozey C, Earl EM (2000). Coactivation of the ankle musculature during maximal isokinetic dorsiflexion at different angular velocities.. Eur J Appl Physiol.

[76] Billot M, Simoneau E, Van Hoecke J, Martin A (2009). Coactivation at the ankle joint is not sufficient to estimate agonist and antagonist mechanical contribution.. Muscle Nerve.

[77] Behm DG, Sale DG (1996). Influence of velocity on agonist and antagonist activation in concentric dorsiflexion muscle actions.. Can J Appl Physiol.

[78] Aagaard P, Simonsen EB, Andersen JL, Magnusson SP, Bojsen-Moller F (2000). Antagonist muscle coactivation during isokinetic knee extension.. Scand J Med Sci Sports.

[79] Kellis E (1998). Quantification of quadriceps and hamstring antagonist activity.. Sports Med.

[80] Maganaris CN, Baltzopoulos V, Sargeant AJ (1998). Differences in human antagonistic ankle dorsiflexor coactivation between legs; can they explain the moment deficit in the weaker plantarflexor leg?. Exp Physiol.

[81] Carmeli E, Reznick AZ, Coleman R, Carmeli V (2000). Muscle strength and mass of lower extremities in relation to functional abilities in elderly adults.. Gerontology.

[82] Hyatt RH, Whitelaw MN, Bhat A, Scott S, Maxwell JD (1990). Association of muscle strength with functional status of elderly people.. Age Ageing.

